# Tripartite collaboration of blood‐derived endothelial cells, next generation RNA sequencing and bioengineered vessel‐chip may distinguish vasculopathy and thrombosis among sickle cell disease patients

**DOI:** 10.1002/btm2.10211

**Published:** 2021-01-09

**Authors:** Tanmay Mathur, Jonathan M. Flanagan, Abhishek Jain

**Affiliations:** ^1^ Department of Biomedical Engineering Texas A&M University College Station Texas USA; ^2^ Department of Pediatrics, Section of Hematology‐Oncology Baylor College of Medicine Houston Texas USA; ^3^ Department of Medical Physiology College of Medicine, Texas A&M Health Science Center Bryan Texas USA

**Keywords:** bioinformatics, organoids and organ‐mimetic systems, tissue engineering

## Abstract

Sickle cell disease (SCD) is the most prevalent inherited blood disorder in the world. But the clinical manifestations of the disease are highly variable. In particular, it is currently difficult to predict the adverse outcomes within patients with SCD, such as, vasculopathy, thrombosis, and stroke. Therefore, for most effective and timely interventions, a predictive analytic strategy is desirable. In this study, we evaluate the endothelial and prothrombotic characteristics of blood outgrowth endothelial cells (BOECs) generated from blood samples of SCD patients with known differences in clinical severity of the disease. We present a method to evaluate patient‐specific vaso‐occlusive risk by combining novel RNA‐seq and organ‐on‐chip approaches. Through differential gene expression (DGE) and pathway analysis we find that BOECs from SCD patients exhibit an activated state through cell adhesion molecule (CAM) and cytokine signaling pathways among many others. In agreement with clinical symptoms of patients, DGE analyses reveal that patient with severe SCD had a greater extent of endothelial activation compared to patient with milder symptoms. This difference is confirmed by performing qRT‐PCR of endothelial adhesion markers like E‐selectin, P‐selectin, tissue factor, and Von Willebrand factor. Finally, the differential regulation of the proinflammatory phenotype is confirmed through platelet adhesion readouts in our BOEC vessel‐chip. Taken together, we hypothesize that these easily blood‐derived endothelial cells evaluated through RNA‐seq and organ‐on‐chips may serve as a biotechnique to predict vaso‐occlusive episodes in SCD patients and will ultimately allow better therapeutic interventions.

## 1 INTRODUCTION

Sickle cell anemia (SCA) along with its other clinical subtypes (sickle cell β thalassemia, hemoglobin SC, etc.) is the most prevalent rare disease in the United States and most common genetic disease in the world.^1^ Roughly 100,000 people are affected in the United States, out of which the African‐American population has a particularly higher incidence of the disease, with at least one individual out of 13 carrying the autosomal recessive mutation.^2^, ^3^ Sickle cell disease (SCD) is characterized by a complex gamut of hematological and vascular complications.^4^ Within the vessels, the unusual vaso‐occlusive cascade involves endothelial activation, platelet adhesion and red cell binding, that can differ among patients. A hypercoagulable state of SCD blood further exacerbates the endothelial–blood interactions and can lead to vaso‐occlusion.^5^ The acute and chronic manifestations of vasculopathy in SCD are multifactorial as they are dependent on the relative hemoglobin distribution, extent of red cell hemolysis, presence of cell‐free hemoglobin and heme, hypercoagulability of blood and endothelial activation.^6^ Also, nearly a quarter of SCD patients encounter a stroke by the age of 45 years,^7^ and the risk of stroke is associated with inherent vasculopathy.

The complications contributing to the vasculopathy in SCD result from a combination of proinflammatory phenotype of the native endothelium and a hypercoagulable state of blood.^8^, ^9^, ^10^ Development of relevant animal models and advancements in the field of in vitro tissue engineered models, like organ‐on‐chip, have greatly enhanced our understanding of the disease.^11^, ^12^, ^13^ However, there is still a considerable knowledge gap in understanding the clinical heterogeneity within the SCD population as these models cannot recapitulate population‐specific outcomes of the disease. It has been observed clinically that different patients show different extents and frequencies of vaso‐occlusive crises,^14^ which ultimately necessitates the need of a predictive model that can differentiate patients and can aid clinicians as a risk evaluation methodology.

An essential requirement for developing a model that mimics patient pathophysiology is to identify autologous cell sources that can recapitulate patient‐specific readouts in vitro.^15^ In our recent work, we have identified blood outgrowth endothelial cells (BOECs) isolated from circulation as a disease‐specific primary cell source to analyze endothelial activation and thromboinflammation in vitro.^16^ We further hypothesize that they can potentially mimic patient‐specific responses in disease. BOECs exhibit classical endothelial characteristics similar to primary cells and can reveal disease‐specific differences in endothelial activation, oxidative stress and metabolic activity relative to control cells, once incorporated in the microfluidic vessel‐chips.^16^ Increased presence of circulating endothelial cells in vascular disorders also makes them a viable cell model.^17^, ^18^, ^19^

Advancements in next‐generation sequencing (NGS) like RNA‐seq has further enabled assessment of differential gene expression in health and disease with high fidelity. Combining the predictive power of autologous, patient‐derived cells like BOECs with tools like RNA‐seq can allow investigation of patient‐specific genome signature. Incorporating BOECs in organ‐ or vessel‐chips can further help in functional validation of patient‐specific phenotype as predicted by RNA‐seq and ultimately lead to development of a patient assessment pipeline.

In this report, we test the aforementioned methodology by isolating BOECs from two patients with known differences in their clinical SCD severity. We explored if easily derived BOECs taken from these patients may serve as: (1) a biomarker to validate the distinct clinical difference between the two patients; and (2)through RNA‐seq analysis to diagnose a potentially differential molecular pathophysiology related to endotheliopathy and thrombosis. Through RNA‐seq and differential gene expression (DGE) studies of these cells, as well as phenotypic assessment through vessel‐chip blood perfusion experiments, we provide a proof‐of‐feasibility of using this integrative approach to assess endotheliopathy and thrombotic potential among SCD patients from tissue‐to‐molecular scale.

## 2 RESULTS AND DISCUSSION

**FIGURE 1:**
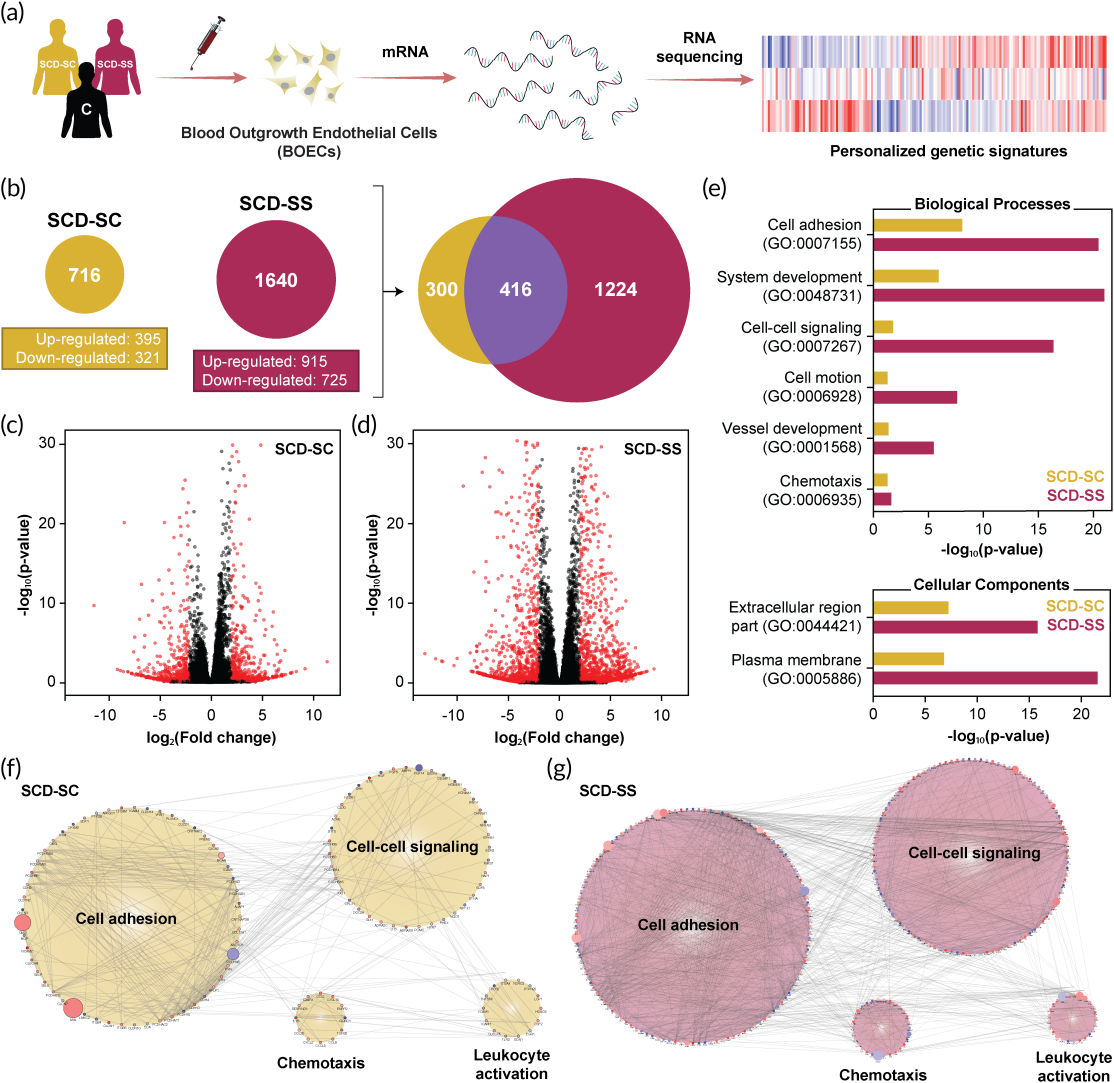
Qualitative assessment of differential gene expression among sickle cell disease patients through autologous BOECs and RNA‐seq. (a) Schematic of the BOEC isolation, expansion and subsequent mRNA extraction process followed in this study. Isolated mRNA from control and SCD patient (SCD‐SC and SCD‐SS). BOECs were then assessed for quality and processed for sequencing. (b) Post‐RNA sequencing and subsequent alignment, differential gene expression analysis revealed significant differences between patient genetic signatures; SCD‐SS BOECs had significantly more differentially expressed genes compared to SCD‐SC. Out of the ~2000 genes analyzed, roughly 400 genes were conserved in SCD‐SC and SCD‐SS. (c and d) Volcano plots of the differentially expressed genes for SCD‐SC and SCD‐SS, respectively, relative to heathy controls. In agreement with (b), SCD‐SS BOECs exhibit relatively higher and statistically stronger fold change differences compared to SCD‐SC (black: excluded genes with −2 < log_2_[FC] < 2, red: differentially expressed genes). (e) Gene ontology (GO) based clustering of differentially expressed genes indicate differences within regulating biological processes and key cellular components between SCD‐SC and SCD‐SS (*p* < 0.05). (f and g) Gene cluster networks exhibiting complex interactions between the most prominent biological processes regulating vascular tone (cell adhesion: GO:0007155; cell–cell signaling: GO:0007267; chemotaxis: GO:0006935, and leukocyte activation: GO:0045321). Compared to SCD‐SC, patient SCD‐SS expressed significantly more genes and hence exhibited more complex gene interactions among the aforementioned biological processes (red, up‐regulated; blue, down‐regulated; size increases with significance)

We initiated the study by selecting two age‐matched patients who represented significantly different clinical manifestations of the sickle cell disease (Table 1). The critical distinction between the two was that one patient had hemoglobin SC disease (SCD‐SC) with a relatively milder disease severity, while the other patient had hemoglobin SS (SCD‐SS) and had a confirmed history of stroke and transfusion therapy, very likely susceptible to endothelial dysfunction and thrombosis.^20^, ^21^ Hemoglobin SC (HbSC) disease is clinically considered a milder variant of SCA although the treatments available to patients are largely derived from studies performed on hemoglobin SS patients.^22^ Although the two subtypes constitute the majority of SCD population with ~30% of patients having the HbSC mutation, the clinical manifestation and phenotype are very different.^23^ Being the less severe phenotype, patient morbidity and mortality are lower among the HbSC patients. On the other hand, patients with sickle cell anemia (1) have more exaggerated inflammatory profiles in blood, (2) have a higher incidence of irreversible RBC sickling, (3) have shortened RBC lifespans compared to hemoglobin SC patients, (4) witness more vaso‐occlusive episodes, and (5) are more susceptible to infections.^24^, ^25^ Reports suggest that HbSC disease patients have lower levels of fetal hemoglobin (HbF) compared to SCA counterpart and the same is witnessed in our findings (Table 1). Hence it is of utmost importance that we gain knowledge of the clinical distinction and possible manifestations to develop better disease management strategies and targeted therapies for the two SCD variants. After selecting the patients, we isolated mRNA from respective patient BOECs and processed them for next generation RNA sequencing **(**Figure 1a **)**. Post‐sequencing and alignment of sequence reads, we investigated differential gene expression among the SCD patients with respect to control BOECs. The DGE results showed that our mild patient (SCD‐SC) had significantly lower number of differentially expressed genes compared to the severe case (SCD‐SS); there were 716 genes differentially regulated in SCD‐SC while SCD‐SS had 1640 genes relative to control (Figure 1b). However, within the gene profiles of the two patients, 416 genes were conserved in both patients implying that these genes might be the prominent regulators of the sickle cell phenotype in patients (Figure 1b). Despite differences in number of genes expressed by the respective patients, SCD‐SS had a greater magnitude of upregulation/downregulation compared to SCD‐SC (Figure 1c,d), indicating that BOECs from SCD‐SS may exhibit a more adverse sickle cell phenotype. Further, the genes unique to SCD‐SS (~1200) might be regulating further downstream endothelial activation and vascular adhesion pathways (Figure S1) that may exacerbate the existing proinflammatory and prothrombotic phenotype.

**TABLE 1:** Complete blood count (CBC) data, clinical history and treatment details of Control and SCD patients

Patient	Age	Sex	WBC	RBC	HbG	HCT	HbF	Platelet	Clinical history	Treatment
Control	23	F	9.23	5.58	15.8	45.7	–	237	–	–
SCD‐SC	10	M	5.38	4.15	10.5	29.5	2	135	Pain with exercise Retinopathy	NA
SCD‐SS	17	F	14.7	3.57	9.8	29.6	5.9	405	Stroke Iron overload Elevated blood pressure	Transfusion

To identify the possible differences in biological responses of the two patients, we performed a gene ontology (GO) enrichment analysis for biological processes (BP), cellular component (CC) and molecular function (MF) GO categories using the online functional annotation tool DAVID (Database for Annotation Visualization and Integrated Discovery).^26^ Between the two patients, the severe SCD‐SS case showed enrichment for total 104 GO terms (*p* < 0.05; 71 for BP, 19 for CC and 14 for MF; Figure S2a), while the mild SCD‐SC case exhibited enrichment for 23 GO terms (*p* < 0.05; 13 for BP, 10 for CC; Figure S2b). Upon narrowing down the GO terms based on high statistical significance (*p*‐value) in each category, we observed that there were significant differences in the enrichment for the most prominent GO terms between the two patients (Figure 1e). Among the patients, the key enriched GO terms for BP were cell adhesion (GO:0007155), system development (GO:0048731), cell–cell signaling (GO:0007267), cell motion (GO:0006928), blood vessel development (GO:0001568) and chemotaxis (GO:0006935), while in CC, plasma membrane (GO:0005886) and extracellular region part (GO:0044421) GO terms were enriched (Figure 1e). Analyzing genes specific to cell adhesion (GO:0007155) suggest that these genes contribute to endothelial activation and thromboinflammation as suggested by the KEGG pathway analysis (Figure S3a). Additionally, these genes are differentially regulated among the two patients with SCD‐SS having a stronger presence of cell adhesion molecule (CAM) and ECM‐receptor interactions contributing to the activated state of these BOECs (Figure S3a,b). The clustering results suggest that among the SCD patient BOECs, biological processes related to endothelial dysfunction/inflammation, are most prominent and are differentially regulated among the two patients, with the severe SCD‐SS case exhibiting higher regulation of endothelial activation relative to the mild SCD‐SC.

To further visualize the differences between the regulation of different biological processes and their related endothelial activation pathways, we generated network clusters for investigating interactions among genes belonging to biological processes regulating endothelial activation (cell adhesion: GO:0007155; cell–cell signaling: GO:0007267; chemotaxis: GO:0006935; and leukocyte activation: GO:0045321) using Cytoscape.^27^, ^28^ As expected, the severe SCD‐SS case had more genes regulating these processes compared to SCD‐SC and exhibited stronger interactions between the regulating genes (Figure 1f,g). This broad categorization of biological processes into the GO terms listed above in fact encompassed few critically suspected endothelial activation and thromboinflammation pathways as predicted by KEGG analysis (Figure S4). Specifically, the family of genes encoding for cell adhesion molecules was upregulated in the patients and contributes to the thromboinflammatory phenotype of these blood derived cells.^29^, ^30^ Taken together, these results support that the SCD patient who had a history of stroke and was clinically diagnosed with severe SCD symptoms, had a transcriptomic upregulation of endothelial activation and thrombosis.

**FIGURE 2:**
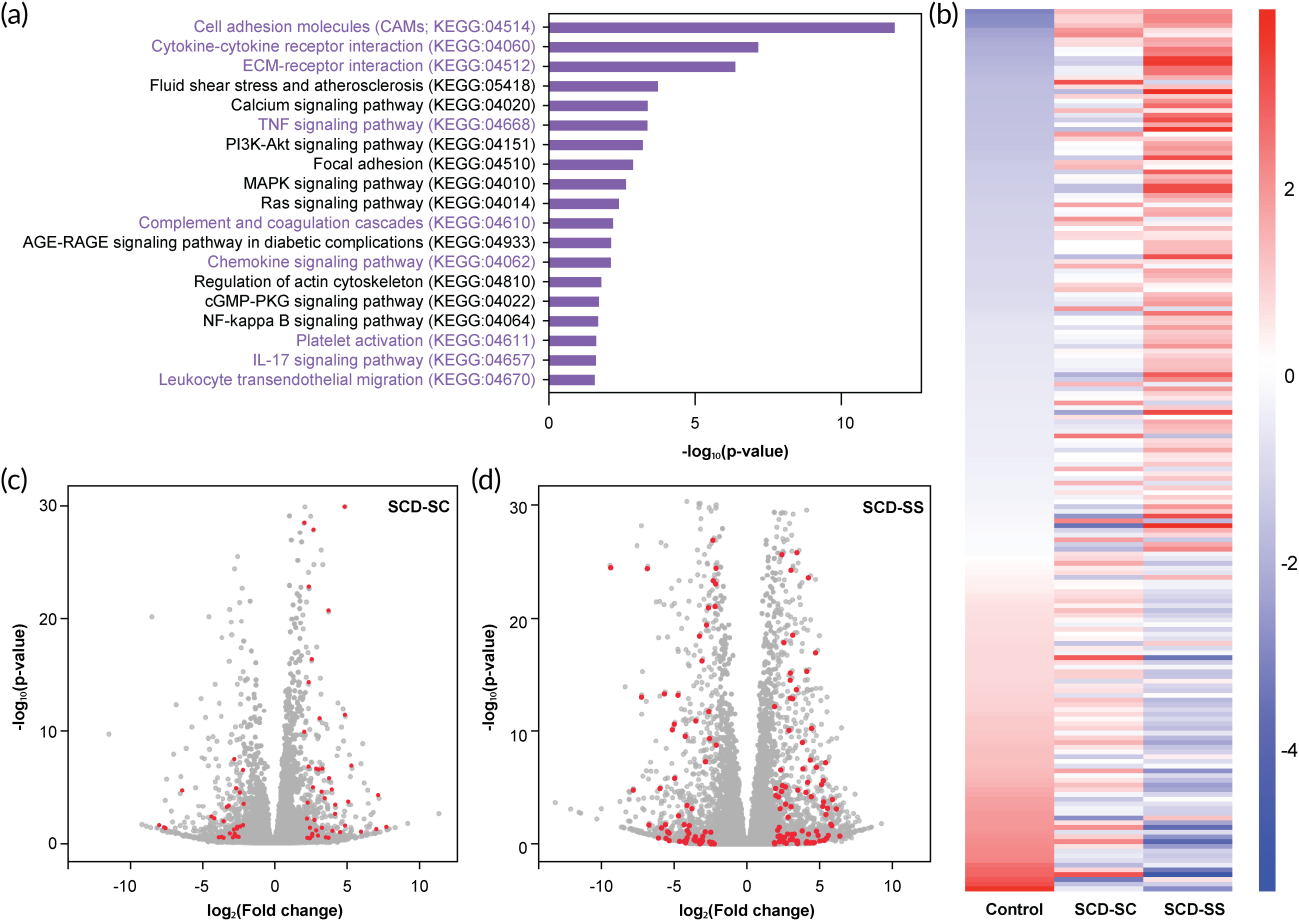
Discerning patient‐specific vascular inflammation and endothelial dysfunction in SCD. (a) KEGG pathway–based clustering indicates prevalence of pro‐inflammatory and pro‐thrombotic processes among other regulatory processes in patients SCD‐SC and SCD‐SS relative to healthy controls (*p* < 0.05). Cell adhesion molecules (KEGG:04514), cytokine–cytokine receptor interaction (KEGG:04060), and ECM receptor interaction (KEGG:04512)are the strongest and most abundant cellular processes observed in SCD‐SC and SCD‐SS. (b) Heatmap depicting row‐scaled *z*‐scores of ~350 genes (sorted w.r.t. control) belonging to cell adhesion molecule, cytokine–cytokine receptor interaction, and ECM receptor interaction families for control, SCD‐SC and SCD‐SS BOECs. Patient SCD‐SS had a nearly complementary gene expression profile compared to control indicating that SCD‐SS BOECs exhibit a dysfunctional and inflammatory phenotype; SCD‐SC on the other hand exhibit a gene profile that is between control and SCD‐SS (*p* < 0.05, red: upregulated; blue: downregulated). (c and d) Genes involved in the aforementioned inflammatory processes are differentially expressed between patients SCD‐SC and SCD‐SS respectively, relative to control (red: respective genes of interest; gray: respective global gene profile)

To further identify the extent of endothelial activation among the patients, we performed a KEGG pathway clustering of the conserved genes (~400, Figure 1b) from the two patient BOECs. Upon clustering, we found that pathways mediating vascular cell–cell signaling through cytokines, cell–cell interactions through adhesion molecules and ECM proteins are the most significant biological pathways that are present in SCD (Figure 2a). Specifically, cell adhesion molecule (CAM; KEGG:04514), cytokine–cytokine receptor interaction (KEGG:04060) and ECM receptor interaction (KEGG:04512) are the most prominent pathways among the patients, while other inflammation pathways like TNF signaling (KEGG:04668), complement and coagulation cascades (KEGG:04610), chemokine signaling (KEGG:04062), platelet activation (KEGG:04611), and leukocyte transendothelial migration (KEGG:04670) pathways were also present (Figure 2a).

To investigate the differential expression of genes belonging to the aforementioned KEGG pathways, we generated heatmaps for comparison among the two patients relative to controls (Figure 2b). Interestingly, BOECs from severe SCD‐SS patient expressed genes contributing to endothelial activation to a higher extent relative to control and SCD‐SC implying that BOECs from SCD‐SS were in a severely thromboinflammation state. In contrast, BOECs from patient SCD‐SC exhibited signs of endothelial dysfunction that were intermediate between that of controls and SCD‐SS (Figure 2b). Such widespread comparison between patients not only revealed the differential presence of these pathways, but also the extent to which they were differentially expressed; SCD‐SS had a much diverse expression profile with more upregulated/downregulated genes, while SCD‐SC had fewer genes being differentially regulated (Figure 2c,d). These results agree with the qualitative gene expression profiles described earlier (Figure 1) as well as the clinical histories of the two patients (Table 1).

**FIGURE 3:**
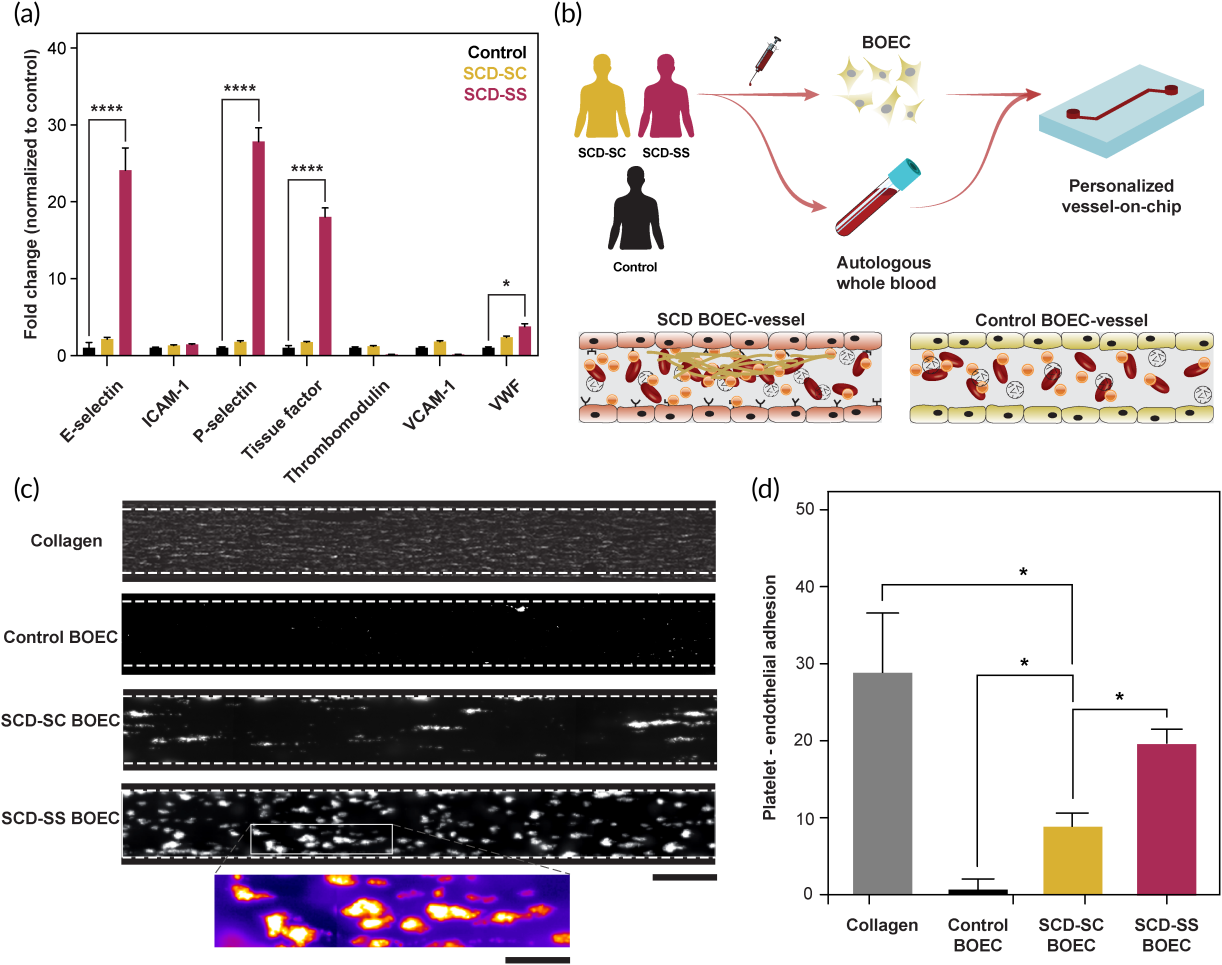
Functional assessment of patient‐derived BOECs and vessel‐on‐chip assembly. (a) Quantification of expression of common endothelial surface markers through qRT‐PCR reveals a significant upregulation in E‐selectin, P‐selectin, tissue factor, and VWF in SCD‐SS BOECs relative to control. In agreement with the sequencing results, SCD‐SC BOECs exhibited lower expression of the aforementioned markers relative to SCD‐SS although more than that of controls. (b) Schematic of the thromboinflammation analysis performed with patient BOECs vessel‐chips. After an overnight culture in rectangular microchannels under constant laminar media perfusion, autologous BOECs were exposed to healthy whole blood in vitro and subsequent platelet adhesion and clotting events were monitored through real‐time fluorescent microscopy. (c) Platelet adhesion micrographs over collagen, control BOEC and SCD BOEC (SCD‐SC and SCD‐SS) vessel‐chips (scale bar: 200 μm). In agreement with our hypothesis and RNA sequencing results, BOECs from SCD‐SS exhibited a more dysfunctional endothelial phenotype in vitro compared to SCD‐SC BOECs, which in turn presented a higher degree of endothelial activation w.r.t. control BOECs . Inset: the classic “comet” morphology of adhered platelets (scale bar: 80 μm). (d) Quantified fluorescence of micrographs showing a significant increase in platelet area coverage for SCD BOEC (SCD‐SC and SCD‐SS) compared to control BOEC vessel‐chips. *p < 0.05, ****p < 0.0001; *n* = 3 for all experiments

In order to support the results obtained through the RNA‐seq and DGE studies, we also analyzed common endothelial activation and vaso‐protective markers like E‐selectin, P‐selectin, ICAM‐1, VCAM‐1, tissue factor (TF), thrombomodulin, and von Willebrand Factor (VWF). Selectins, specifically P‐selectin, have been implicated in SCD causing endothelial‐RBC interactions and subsequent thrombosis and ischemia.^12^, ^15^ Tissue factor expression by endothelial cell in SCD physiology has also been postulated to contribute to the ensuing vaso‐occlusive crises.^31^ In agreement with these findings, our results reveal that among the common adhesion proteins expressed by the endothelium, both SCD patients had an upregulation of E‐selectin, P‐selectin, tissue factor, and VWF while other markers like ICAM‐1 and VCAM‐1 were moderately upregulated (Figure 3a). Additionally, these genes were differentially regulated between the two patients with SCD‐SS exhibiting a higher fold change expression compared to SCD‐SC and both patients having more expression than control (Figure 3a). Taken together, these results suggest that RNA‐seq of BOECs from SCD patients may serve as a model to assess SCD patient severity.

Finally, we set out to investigate phenotypic differences that the BOECs exhibit between the SCD patients and predict microvascular thromboinflammatory consequences due to disease severity within the patients. Our prior work has repeatedly shown that in vitro blood vessel organ‐on‐a‐chip is a platform technology to visualize blood–endothelial interactions in real‐time.^32^, ^33^ Hence, we created microfluidic vessel‐chips lined with BOECs on all sides of a hollow matrix‐coated microfluidic chamber. Once these BOEC “blood arterioles” were ready, we perfused them with autologous blood samples at arteriolar flow conditions and examined real‐time platelet–endothelial adhesion and coagulation using fluorescence microscopy (Figure 3b, Movie S1). We observed that BOEC‐vessel‐chip of the SCD patients were both more adhesive than normal controls. However, severe SCD‐SS patient had a significantly higher platelet adhesion to the BOEC endothelium, relative to the mild SCD‐SC patient, demonstrating that BOECs of a severe SCD case are hyperactivated and prothrombotic (Figure 3c,d and Movie S1). These functional blood perfusion studies also correlate to the DGE results obtained through RNA‐seq (Figures 1 and 2) and suggest that harnessing BOECs from patient blood samples, and analyzing them through RNA‐seq and vessel‐chips may provide a genotype and phenotype signature potentially valuable in assessing disease severity in SCD.

## 3 CONCLUSIONS

In this proof‐of‐concept study, we present a patient vaso‐occlusive risk assessment methodology utilizing a novel combination of autologous endothelial progenitors from cardiovascular patients as an alternative cell model, RNA‐sequencing and organ‐on‐chip technology. Our results suggest that autologous cells like BOECs can be effective in providing the state of endothelial health and might be predictive of a patient's in vivo pathophysiology. The ability of autologous BOECs to mimic a patient's native endotheliopathy can further allow clinicians to phenotype patient‐to‐patient variation in disease severity. Additionally, studies report that circulating endothelial progenitors like BOECs are increased in cardiovascular patient circulation compared to healthy individual thereby further bolstering their use as an alternate cell model.^17^, ^19^ Although we have chosen sickle cell disease as a model to test our hypothesis that BOECs recapitulate patient‐specific endotheliopathy in vitro, this approach can potentially be applied to other cardiovascular complications like atherosclerosis,^34^ diabetes,^35^, ^36^ thrombosis^37^, ^38^ and other conditions that witness significant endothelial activation and vascular inflammation.

In agreement with clinical findings that patients with HbSC disease indeed have lower extents of vaso‐occlusive episodes compared to SCA patients and exhibit milder disease severity, we demonstrate such differences in gene expression profiles which we then correlate to the functional blood perfusion readouts using organ‐chips as well as with the patient clinical history available. The blood perfusion experiments elicit differences in endothelial–blood interaction between the two SCD subtypes and this difference is further validated by quantifying relevant endothelial activation markers like E‐selectin, P‐selectin, VWF, and tissue factor.

Current in vitro microfluidic models of SCD have put primary focus on red blood sickling and hemolysis in SCD and the endothelial activation in SCD has been relatively understudied.^39^, ^40^ As a result, there is a knowledge gap in understanding the interactions between native endothelium and blood components in SCD microcirculation. Inability to study the convoluted transformation from a healthy, to an “activated” state and ultimately acquiring a “dysfunctional” endothelial phenotype has added additional burden over existing disease management strategies. Previously published studies have reported endothelial–blood interactions in SCD, they however utilize primary cells isolated from healthy individuals that are exogenously stimulated to mimic an activated endothelium and hence cannot elicit differences in endothelial–blood crosstalk among patients.^41^, ^42^ Consequently, this is a first‐of‐its‐kind study utilizing autologous SCD patient cells to characterize differential vascular dysfunction between two clinically diverse patients.

We compare the gene expression profiles of these patients and categorize the differentially expressed genes into biological processes and molecular pathways using widely used pathway annotation tools like DAVID and Cytoscape that offer gene ontology (GO) and KEGG pathways–based clustering. Although these annotation methodologies have their caveats as clustering is often broad, specificity can be low and matching of pathways is limited to the current annotations present in the database,^43^, ^44^ these can still provide holistic differences between patient genetic profiles.

**FIGURE 4:**
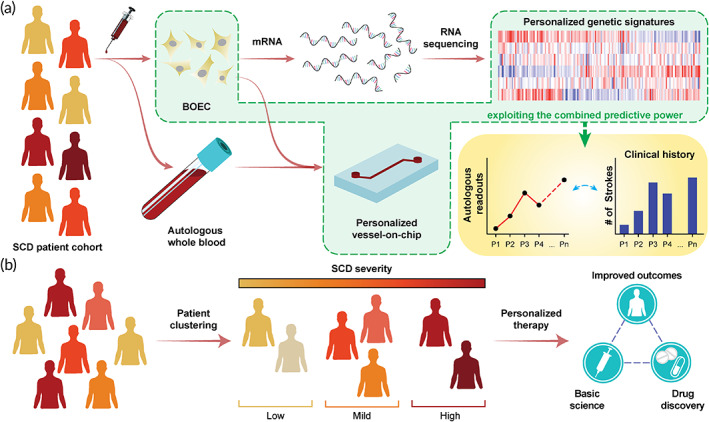
Future scope of the BOEC–RNA‐seq–organ‐chip pipeline to evaluate patient‐specificity within SCD population. (a) Schematic demonstrating application of the study pursued in this article to a wider, more diverse population of SCD patients. Profiling personalized genetic signatures through RNA‐seq and phenotyping microvascular behavior using organ‐chip technology can allow clinicians and pharmaceutical researchers correlate patient clinical outcomes and eventually improve treatment prospects via personalized therapy. (b) We hypothesize that exploiting the predictive power of BOECs, harnessed via RNA‐seq and organ‐chip technology, can also allow clustering/grouping of patients into different categories based on disease severity. This will enable clinicians to prescribe treatments specific to patient categories, improve the drug discovery and screening pipeline in the pharmaceutical industry, improve therapeutic outcomes and ameliorate patient conditions, and ultimately progress the current state of healthcare

Although we have limited the scope of this study to characterize two patients only, this proof‐of‐feasibility study further lays the groundwork for assessment of a much diverse and extensive SCD patient cohort (Figure 4a). Amalgamation of autologous BOECs with RNA‐seq and microphysiological assessment tools like organ‐chips may yield clinical tools with high predictive power, that can ultimately enable clinicians in identifying individuals at high risk of stroke or cardiovascular complications. The proposed methodology can also be useful in grouping patients into broader groups based on disease severity that can potentially aid pharmaceuticals and clinicians in developing alternative therapeutic strategies and further the scope of personalized medicine (Figure 4b). Although we focus our assessment strategy on SCD in this work, this methodology can also be potentially extended to assess patients with other cardiovascular complications.

## 4 MATERIALS AND METHODS

### 4.1 BOEC isolation and expansion

BOEC colonies were prepared from healthy volunteers (H) and our two SCD patients (SCD‐SC and SCD‐SS) according to the protocol published by our group and others.^16^, ^45^, ^46^ BOECs from circulating patient blood were isolated by collecting 60–100 ml peripheral blood from the SCD and control patients in tubes containing 3.2% sodium citrate. The blood samples were then diluted with 1X PBS (Gibco) in a 1:1 ratio. The diluted blood was then carefully added into 50 ml tubes (Falcon) containing 15 ml density gradient centrifugation media (Ficoll‐Paque PLUS, GE Healthcare). The tubes were inclined such that the angle between the tube and horizontal was ~20° to prevent any mixing of blood with the density gradient centrifugation medium. The tubes were then centrifuged at 400*g* without break and acceleration for 35 min and the distinct “buffy” layer of peripheral mononuclear cells was collected. The total cells were counted using a hemocytometer and plated onto culture flasks precoated with collagen. The cells were supplemented with BOEC growth media (20% fetal bovine serum in EGM‐2, Promocell). The flasks were placed in a standard 37°C incubator with 5% CO_2_. The cell media was replaced every 48 h for 3 weeks until BOEC outgrowth colonies appeared. Once the cell outgrowth colonies reached ~1000 cells/colony, the cells were subcultured and transferred to a new culture flask and cultured until confluent.

### 4.2 RNA sequencing and analysis

BOECs in T25 cell culture flasks with up to 70% confluence were detached from the flasks using 0.05% Trypsin/EDTA. The trypsin solution was neutralized by adding an equal volume of BOEC growth media. The cells suspensions were then centrifuged at 300*g* for 5 min and the supernatant was aspirated. Once collected, total mRNA was extracted from patient BOEC samples using Monarch® Total RNA Miniprep kit (New England Biolabs) following the manufacturer's protocol. The extracted RNA quality were assessed by measuring the spectrometer absorbance ratios between 260/280 nm. Only samples with ratios greater or equal to 2 were used. The patient samples were then analyzed using the NextSeq 500 platform (Illumina) with sample preparation using TruSeqRNA sample preparation and paired‐end read length of 2 × 150 bases (Molecular Genomics Workspace, Texas A&M University, College Station, TX). Following sequencing, raw paired‐end reads (2 × 150) were checked for sequencing adapters and primers with Trimmomatic (v0.38). First, low quality bases were removed using a 25‐bp slide‐window and trimming when the average quality score was below 30 (bases with error probabilities higher than 0.001) and retaining only reads that are of length 125 bp or more. Then we used STAR to splice align the reads to latest ENSEMBL‐release‐98 human genome/transcriptome (GRCh38.p13). For the control group: 45469265, 52441747, 49068269, and 42724819 sequenced reads were successfully aligned to the genome for each of the replicates respectively. Similarly for SCD‐SC: 45469265, 52441747, 49068269, and 42724819; and for SCD‐SS: 53385573, 47839380, 44650747, and 39798937 sequenced reads were successfully aligned to the genome for each of the replicates respectively. The Bioconductor package SUBREAD was used to generate raw counts and differentially expressed genes were evaluated using DESeq2. All analyses were done in R®. The cutoff to determine significant genes in both groups were −2 < log_2_FoldChange < 2 and FDR adjusted *p*‐value (*q*‐value) < 0.05. The GO enrichment analysis was performed using the online database DAVID (Database for Annotation Visualization and Integrated Discovery, v6.8). Visualization of the gene networks was performed using Cytoscape, GeneMANIA and the KEGG pathway analysis was performed using ClueGO.^28^

### 4.3 qRT‐PCR

For qRT‐PCR, the cells were cultured in T25 flasks and their total RNA were isolated using the method described above. Following RNA extraction and quality assessment, cDNA for each patient sample were generated from 1 μg of precursor RNA via ProtoScript® First Strand cDNA Synthesis Kit (New England Biolabs) following manufacturer's protocol. Primers were designed using FASTA sequences for each gene (NCBI Gene database) and ThermoFisher's OligoPerfect. The primers used for the study were as follows: GAPDH (forward: 5′‐GCCAACGTGTCAGTGGTGGA‐3′; reverse: 5′‐CCATGTGGGCCATGAGGTCC‐3′), ICAM‐1 (forward: 5′‐TATGGCAACGACTCCTTCT‐3′; reverse: 5′‐CATTCAGCGTCACCTTGG‐3′), VCAM‐1 (forward: 5′‐ATGACATGCTTGAGCCAGG‐3′; reverse: 5′‐GTGTCTCCTTCTTTGACACT‐3′) E‐selectin (forward: 5′‐ACCTCCACGGAAGCTATGAC‐3′; reverse: 5′‐TCCCAGATGAGGTACACTGA‐3′), Tissue factor (forward: 5′‐CAGACAGCCCGGTAGAGTGT‐3′; reverse: 5′‐CCACAGCTCCAATGATGTAGAA‐3′), VWF (forward: 5′‐ACCGAGACCTGGCAGTATCT‐3′; reverse: 5′‐TGCTGCCTGAGATTCACTGG‐3′), P‐selectin (forward: 5′‐GCAGAAGGCAGAAAACCAGC‐3′; reverse: 5′‐GGGAGGGTCAAAGTGGACAG‐3′), thrombomodulin (forward: 5′‐CAAGAAGTGTCTGGGCTGGG‐3′; reverse: 5′‐GACCCCAAGCATGTTACCCA‐3′). To quantify the amplification we used SYBR green.

### 4.4 Vessel‐chip design and fabrication

Microfluidics vessel‐chips were designed in SolidWorks so that they resembled small arterioles (~100 μm). The vessel channels were 200 μm wide and 75 μm high; this corresponded to a hydraulic diameter of ~110 μm. The design was then patterned on silicon wafers (University Wafer Corp) via soft lithography of PDMS. To allow for fluid flow. Inlet and outlet holes were punched using a 1.5 mm biopsy punch (Ted Pella). Each device had two individual vessels to allow for easy experimental replication. The PDMS slab containing the features was then bonded to a PDMS coated glass slide (75 × 25 mm) using a 100 W plasma cleaner (Thierry Zepto, Diener. Electronic). An open slip‐tip syringe was connected to the channels via a curved dispensing tip (Qosina) that acted as a fluid reservoir. The channels were then connected to a syringe pump (Harvard Apparatus, PHD Ultra) through the outlet using a 20″ tubing (Qosina).

### 4.5 Vessel‐chip functionalization and endothelial cell culture

The microfluidic devices were first treated oxygen plasma for 30 s at a power of 50 W prior to treatment with a 1% solution of (3‐aminopropyl)‐trimethoxysilane (APTES, Sigma‐Aldrich) in 200 proof ethanol. After a 10 min silane treatment, the solution was rinsed out first with 70% ethanol followed by 100% ethanol. The devices were then stored in a 70°C oven for 2 h. The devices were then filled with a 100 μg/ml solution of type‐1 rat tail collagen (Corning) and incubated at 37°C in a 5% CO_2_ incubator for an hour. The collagen solution was then rinsed out with BOEC media. Patient BOECs were trypsinized from confluent cell culture flasks and resuspended BOEC growth media at a concentration of 10 million cells/ml and seeded into pretreated microfluidic devices. The BOEC seeded microfluidic devices were incubated at 37°C in a 5% CO_2_ incubator for an hour. After initial attachment of cells on one side of the microfluidic devices, the process was repeated by seeding a freshly prepared cell suspension at the aforementioned concentration in the devices and incubating again at 37°C in a 5% CO_2_ incubator for an hour while upside‐down to promote BOEC attachment on all sides of the microfluidic channel. To mimic the native vessel physiology, BOEC seeded microfluidic devices were constantly perfused with growth media overnight. The devices were then connected to a syringe pump that perfused BOEC growth media through the devices at flow rate of 1 μl/min (shear stress: 0.81 dynes/cm^2^; shear rate: 81 s^−1^) overnight. This flow rate was chosen to provide the cells with an arteriolar shear rate while optimizing growth media usage.^38^, ^47^, ^48^ Perfusion of growth media ensured constant supply of nutrients to the cells and alignment of the cells along the direction of flow.

### 4.6 Blood perfusion and microscopy

Blood from healthy donors was collected in 3.2% sodium citrate tubes (BD Biosciences) and used according to the Institutional Review Board (IRB) guidelines (IRB ID: IRB2016‐0762D). To ensure consist results and to avoid abnormal coagulation activity, the blood samples were used within 4 h of withdrawal. Five hundred micro liters of blood sample was incubated with FITC‐conjugated anti‐human CD41 antibody (10 μl/ml blood Invitrogen) and fluorescently labeled fibrinogen (20 μg/ml blood, Invitrogen). The labeled blood was then added to an inlet reservoir connected to the endothelialized vessel‐chips and blood was perfused at a flow rate of 15 μl/min which yielded a shear rate of 750 s^−1^. To reinstate coagulation, a solution of 100 mM CaCl_2_ and 75 mM MgCl_2_ was mixed with blood in a 1:10 ratio prior to perfusion.^32^ The devices were mounted on an automated microscope (Ziess Axio Observer) and real‐time fluorescence imaging was performed for a duration of 15 min.

### 4.7 Statistics

All data shown are mean ± SD. Statistical analysis has been performed using GraphPad Prism ver. 8. Comparisons between groups made using ANOVA or Student's *t*‐test. Differences are considered statistically significant if *p* < 0.05.

### 4.8 Study approval

All experiments were performed in accordance with the policies of the US Office of Human Research Protections (OHRP) and Texas A&M University Human Research Protection Program (HRPP). The study was approved by the Texas A&M University Institutional Review Board (IRB ID: IRB2016‐0762D).

## AUTHOR CONTRIBUTIONS

Tanmay Mathur and Abhishek Jain designed the experiments, analyzed data and wrote the manuscript., with support from Jonathan M. Flanagan. Tanmay Mathur fabricated the devices, cultured the cells and performed the experiments. Jonathan M. Flanagan isolated and provided BOECs.

### PEER REVIEW

The peer review history for this article is available at https://publons.com/publon/10.1002/btm2.10211.

## Supporting information

**Data S1**: Supporting Information.

**Movie S1** Time series showing platelet adhesion on BOEC‐vessel‐chips for control and patients SCD1 and SCD2. Each frame is 4 min apart but for presentation, the movie runs at 0.5 frames per second.

## Data Availability

The data that support the findings of this study are available from the corresponding author upon reasonable request.
