# Supplementary material for: Tripartite collaboration of blood‐derived endothelial cells, next generation RNA sequencing and bioengineered vessel‐chip may distinguish vasculopathy and thrombosis among sickle cell disease patients
Source: Bioeng Transl Med. 2021 Jan 9;6(3):e10211. doi: 10.1002/btm2.10211 (PMC8459595; doi:10.1002/btm2.10211)
Supplement: Supplementary file 1 — Data S1: Supporting Information. [file BTM2-6-e10211-s002.docx]

**Tripartite collaboration of blood-derived endothelial cells, next generation RNA sequencing and bioengineered vessel-chip may distinguish vasculopathy and thrombosis amongst sickle cell disease patients**

Tanmay Mathur^1^, Jonathan M. Flanagan^2^ and Abhishek Jain^1,3*^

**SUPPLEMENTARY FIGURES**

**
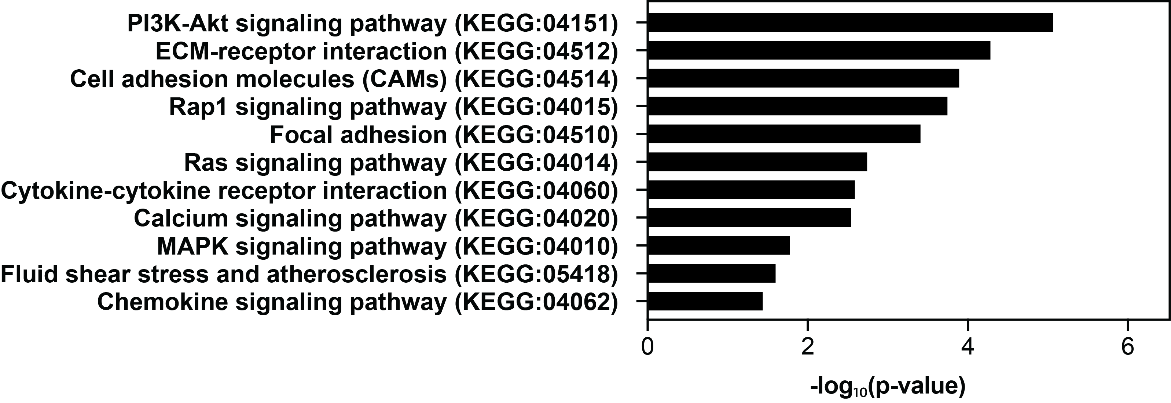
**

**Supplementary Figure 1: KEGG pathway analysis of endothelial activation and thromboinflammation pathways associated with SCD-SS only genes.** Clustering the genes belonging to SCD-SS patient only (~1200) based on KEGG annotation yield pathways that regulate endothelial cell activation and inflammation. Among the list of pathways, PI3-Akt signaling (KEGG:04151), ECM-receptor interaction (KEGG:04512) and Cell adhesion molecules (KEGG:04514) pathways are most prominent.

**
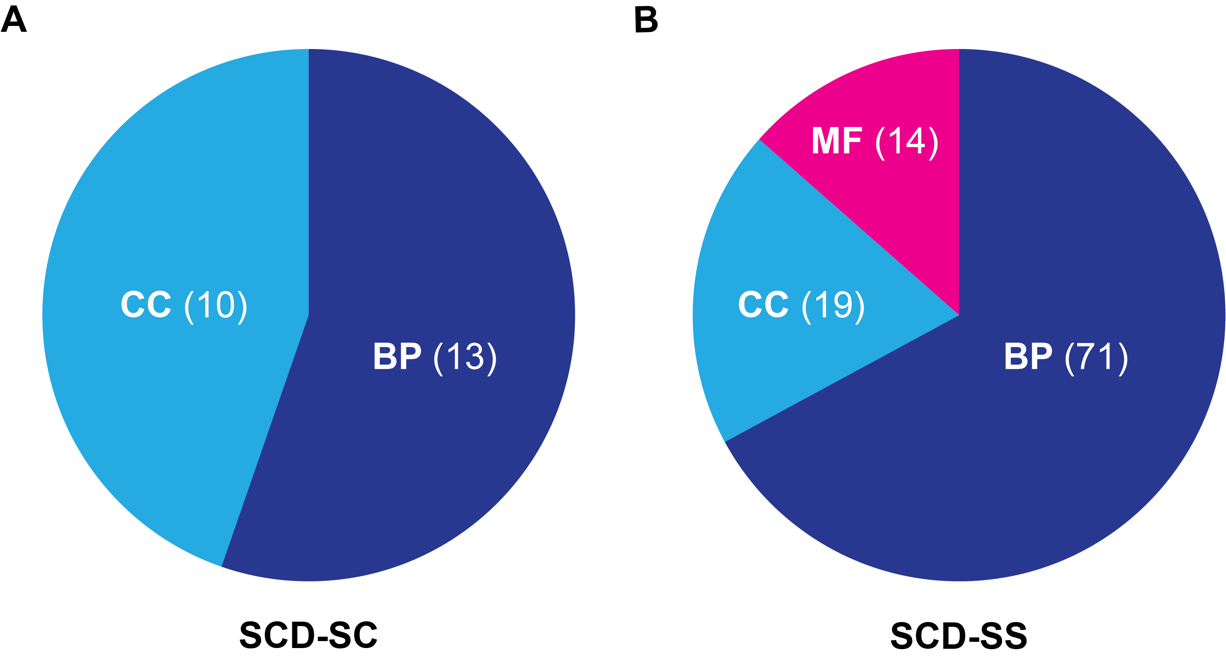
**

**Supplementary Figure 2: Total GO terms enhanced for the SCD patients relative to control.** (A) SCD-SC exhibited enrichment of total of 23 GO terms; 13 for biological processes and 10 for cellular components. (B) On the other hand, SCD-SS exhibited enrichment of total of 104 GO terms; 71 for biological processes, 10 for cellular components and 14 for molecular function.

**
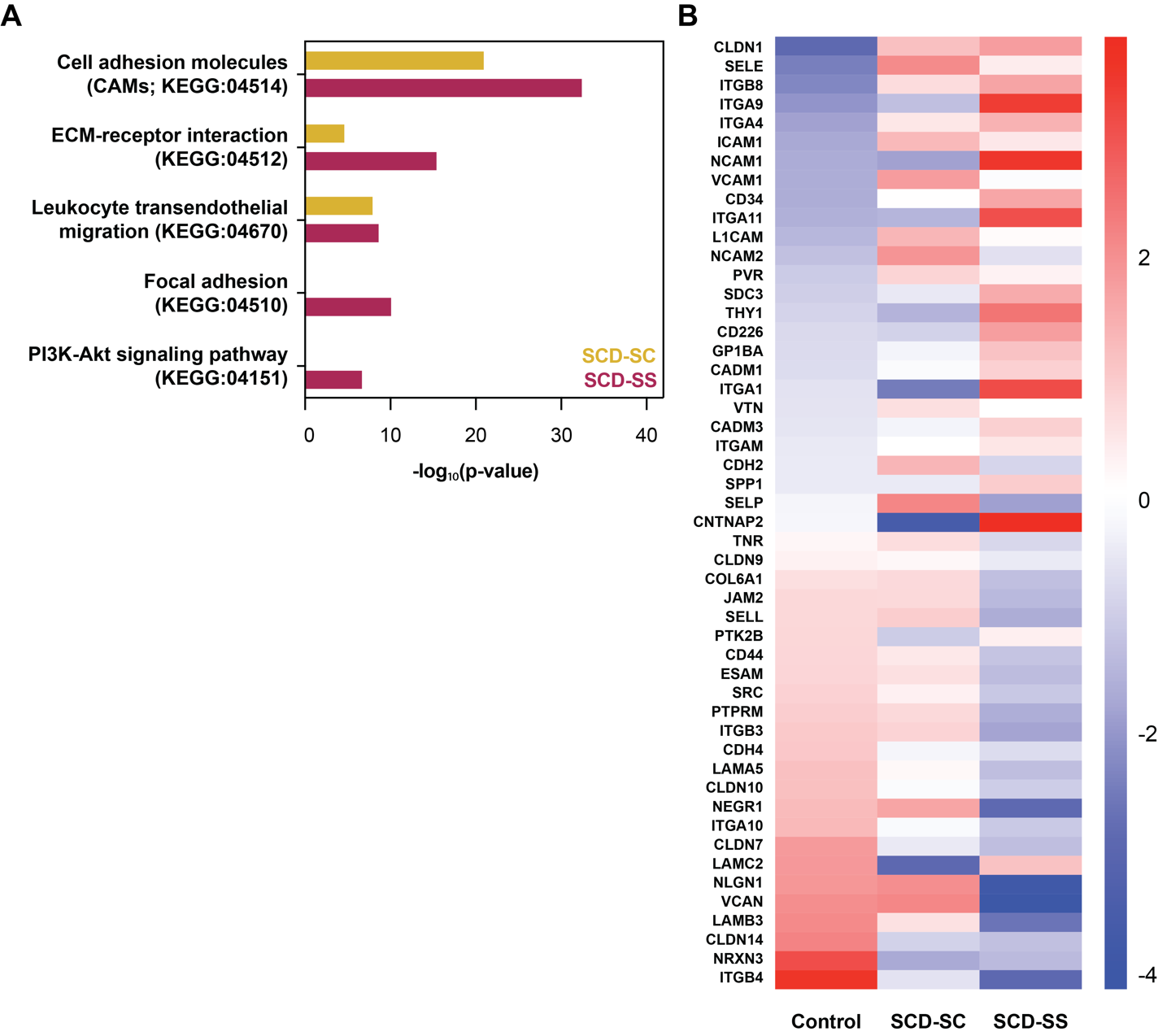
**

**Supplementary Figure 3: KEGG pathway annotation for genes belonging to cell adhesion (GO:0007155).** (A) Pathway annotation of genes belonging to the biological process, cell adhesion (GO:0007155) indicates presence of cell adhesion molecules (KEGG:04514), ECM-receptor interaction (KEGG:04512), leukocyte transendothelial migration (KEGG:04670), focal adhesion (KEGG:04510) and P13-Akt signaling (KEGG:04151) pathways for SCD-SS. Contrastingly, patient SCD-SC only shows enrichment of first 3 of the aforementioned pathways and to a lower extent compared to SCD-SS. (B) Heatmap showing the row-scaled z-scores of ~50 genes (sorted w.r.t. control) belonging to the biological process, cell adhesion (GO:0007155) for control, SCD-SC and SCD-SS BOECs.

**
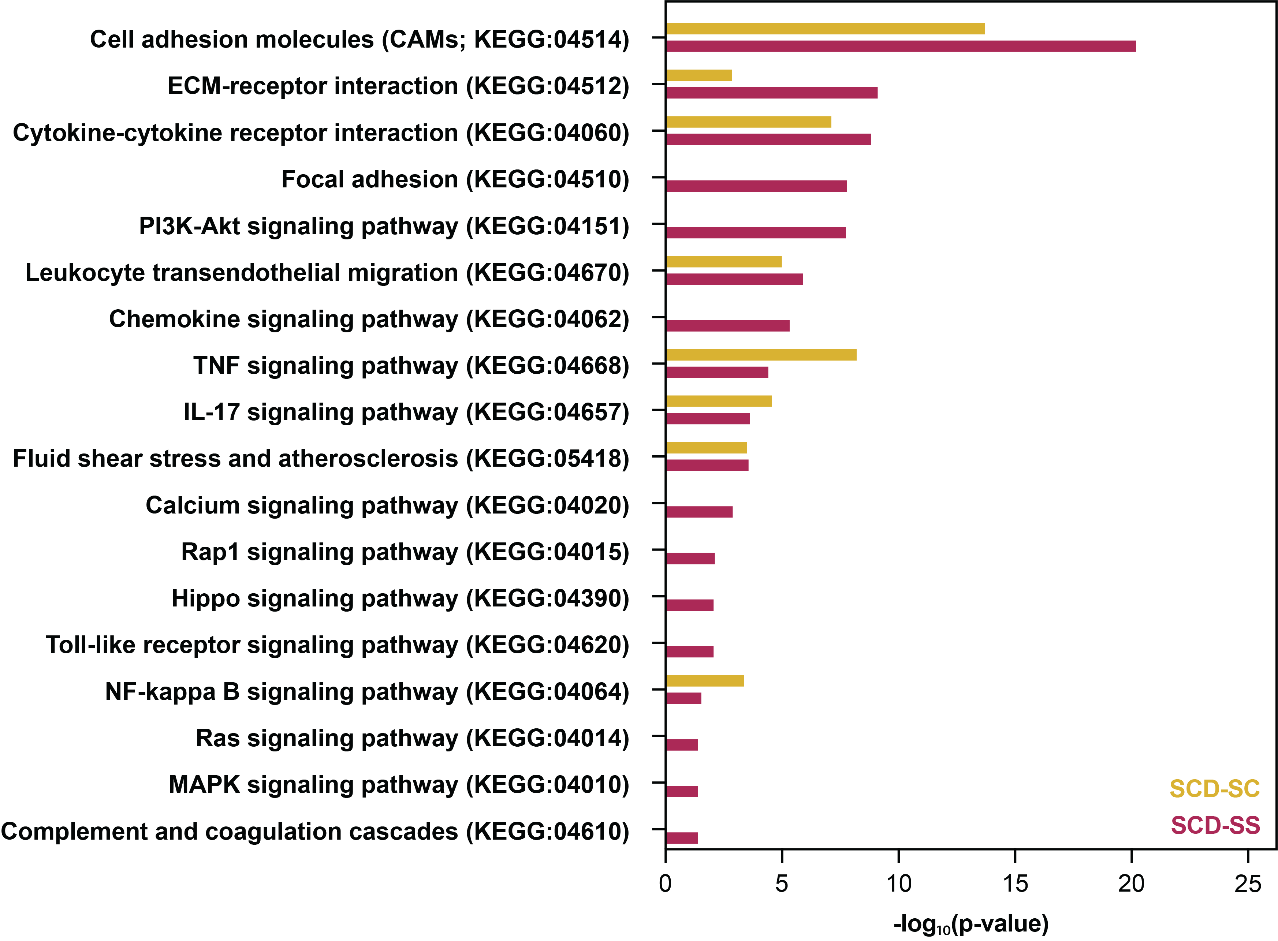
**

**Supplementary Figure 4: KEGG pathway analysis of gene network clusters involving cell adhesion (GO:0007155); cell-cell signaling (GO:0007267), chemotaxis (GO:0006935) and leukocyte activation (GO:0045321) gene ontologies.** KEGG pathways annotation of genes belonging to the network clusters of cell adhesion (GO:0007155); cell-cell signaling (GO:0007267), chemotaxis (GO:0006935) and leukocyte activation (GO:0045321) as shown in Fig. 1F, G. SCD-SS patient exhibited a stronger presence of endothelial activation and thromboinflammatory pathways compared to SCD-SC. This analysis shows that the network clusters in Fig. 1F and G encompass pathways that give SCD BOECs an activated phenotype.

**Supplementary Movie 1:** Time series showing platelet adhesion on BOEC-vessel-chips for control and patients SCD1 and SCD2. Each frame is 4 minutes apart but for presentation, the movie runs at 0.5 frames per second.
